# Extensive clostridial myonecrosis after gluteal intramuscular injection in immunocompromised patient treated with surgical debridement and negative-pressure wound therapy

**DOI:** 10.1016/j.tcr.2021.100469

**Published:** 2021-03-18

**Authors:** Laurine Paquier, Jakov Mihanović, Antoine Counil, Robert Karlo, Ivan Bačić, Boris Dželalija

**Affiliations:** a School of Medicine, University Claude Bernard Lyon, France; b Department of Surgery, Zadar General Hospital, Croatia; c Department of Health Studies, University of Zadar, Croatia; d Department of Infectious Diseases, Zadar General Hospital, Croatia

**Keywords:** *Clostridum perfringens*, Gas gangrene, Intramuscular injections, Negative-pressure wound therapy, Steroids, Immunosuppression

## Abstract

Gas gangrene is infectious disease caused by *Clostridium perfringens* infection.

We are presenting extremely rare case of gluteal clostridial myonecrosis after intramuscular injection of diclofenac in immunocompromised young patient on a long-standing corticosteroid therapy presented with sepsis and initially absent clinical signs of severe anaerobic infection. After delayed diagnosis, she was treated with aggressive surgical removal of necrosed tissue and targeted antibiotic therapy which led to a rapid improvement allowing application of a negative-pressure wound therapy device with favorable outcome.

This report shows the importance of timely diagnosis with pitfalls of imaging. It confirms that surgical debridement along with specific antibiotic therapy is the mainstay of treatment, but also promotes negative-pressure wound therapy which has proved convenient for accelerated closure of large incisions with tissue loss without any adverse effects or the need for complex reconstructive procedures.

## Introduction

Historically gas gangrene was highly lethal condition observed by despondent war surgeons all over the world's battlefields. In most of the cases the cause is wound inoculation of *Clostridium perfringens* (CP), a gram-positive spore forming bacillus. CP is present in natural environment, but also as a commensal of human intestinal and genital tract. It produces toxins which cause tissue necrosis, hemolysis and organ failure syndrome. Consequences of the sepsis and necrosis can lead to amputation of the limbs, extensive tissue loss and even death. It is crucial to make a diagnosis as soon as possible in order to provide adequate surgical and antibiotic therapy.

## Case presentation

**Fig. 1:**
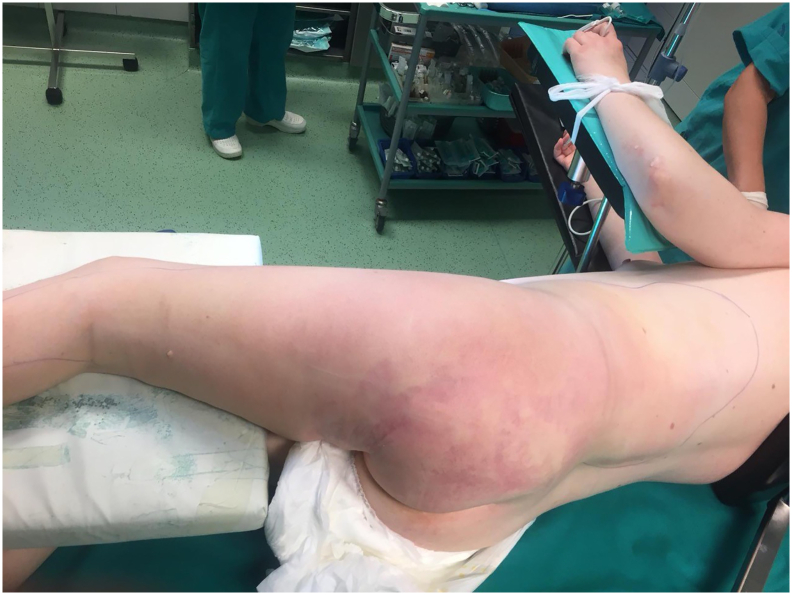
Preoperative photograph of the patient with pen mark showing the extent of redness and swelling.

**Fig. 2:**
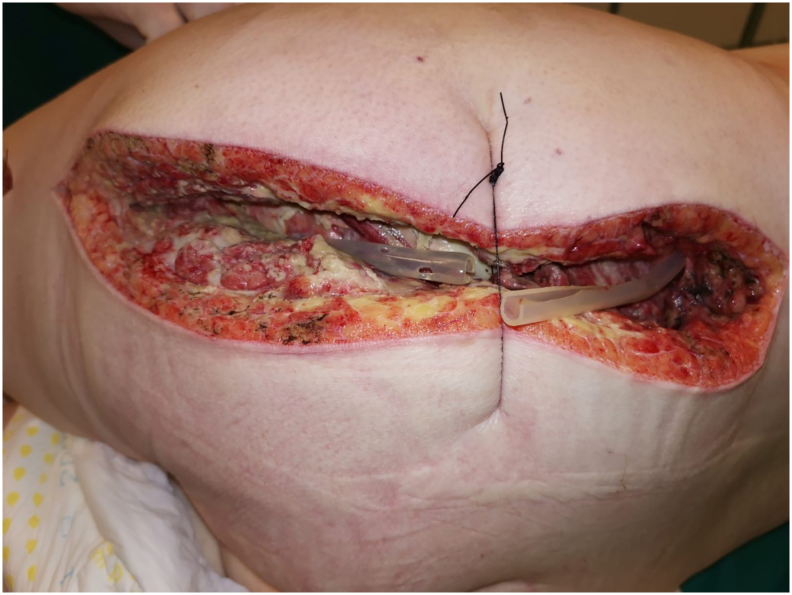
Post-operative wound after debridement and drainage.

**Fig. 3:**
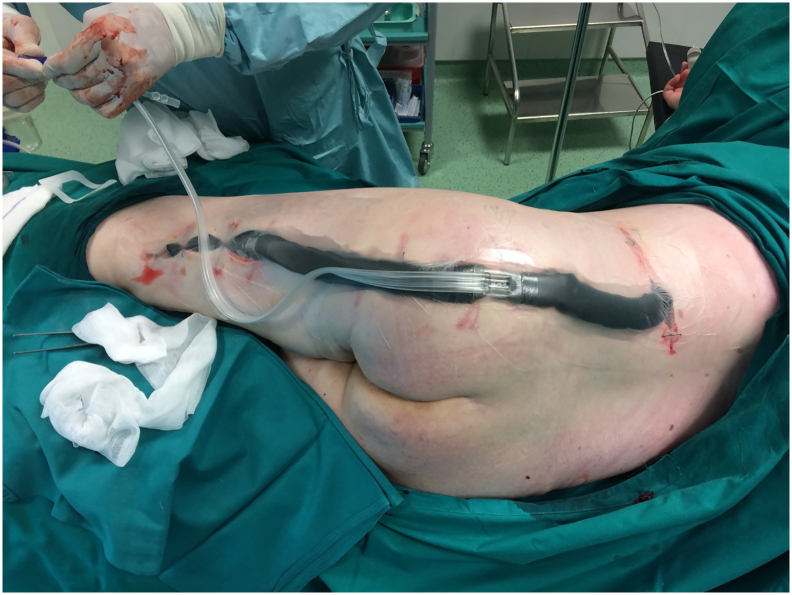
Initial NPWT application.

**Fig. 4:**
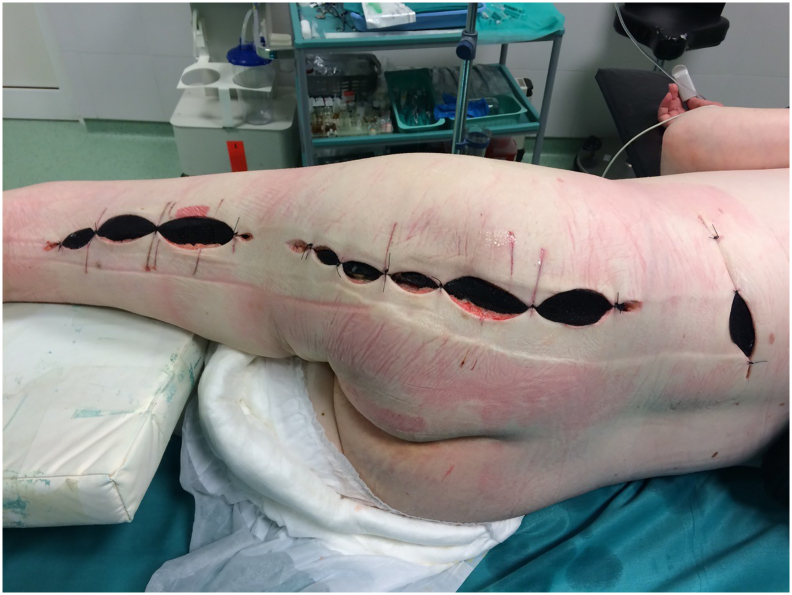
Secondary sutures with NPWT sponges in-between.

**Fig. 5:**
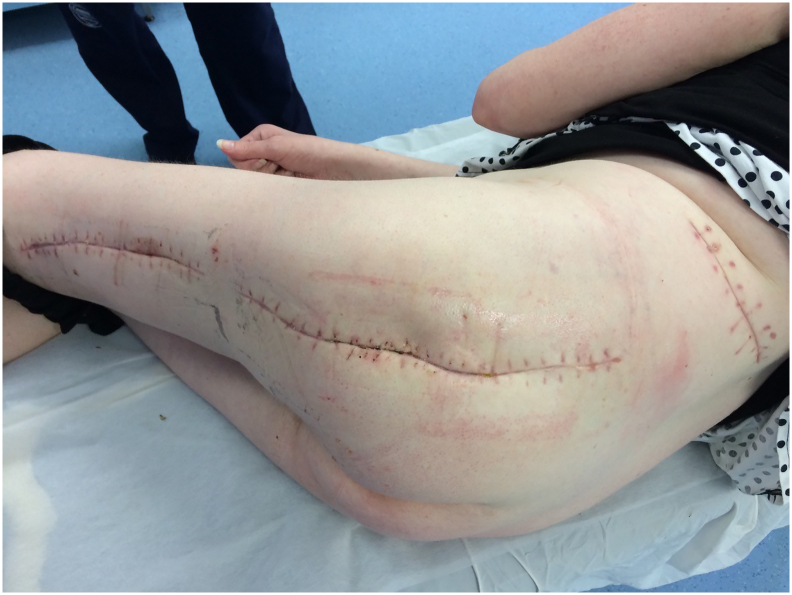
Final appearance after removal of sutures.

33-year-old woman was admitted to the Emergency Room because of general weakness and temperature 39.3 °C with shivers. Two days before admission she complained of lower back pain concerted with her menstrual cycle, therefore she received intramuscular injection of diclofenac 75 mg at general practitioner's office. Patient's medical history included long term corticosteroid therapy for systemic lupus erythematosus and partial thyroidectomy for hyperthyroidism. At admission patient was alert and oriented, tachypneic (30 respirations/min), tachycardic (110 beats/min) and hypotensive (80/60 mmHg) with peripheral oxygen saturation of 98%. Her facial skin was erythematous, her lips were pale and tongue was dry. Chest and abdominal physical exam were unremarkable. Limbs were not swollen. Patient's initial blood tests showed severe sepsis without obvious source. White blood cell count was 37.5 × 10^9^/L with strong left shift (18% of non-segmented neutrophils) and mild anemia with hemoglobin level of 10.6 g/dL. Biological markers of infection were elevated: C-reactive protein (CRP) level 109 mg/L and procalcitonin 16 ng/mL. Renal function was slightly impaired with serum creatinine level 124 mmol/L. Liver function tests were elevated with mild coagulopathy: aspartate transaminase 205 U/L, alanine transaminase 193 U/L, international normalized ratio 1.37, and activated partial thromboplastin time 37 s. Chest X-ray was unremarkable. Urine test showed no signs of infection. The patient was admitted to Department of Internal medicine where broad spectrum antibiotics (amoxicillin/clavulanic acid and ciprofloxacin) were administered intravenously. The same evening her general condition deteriorated with progression of dyspnea urging referral to Intensive Care Unit (ICU). During the following three days her general and laboratory condition showed insignificant improvement. Hemocultures obtained before the start of antibiotic therapy were sterile. The patient started to complain of pain in her left gluteal region where redness and swelling developed. Ultrasound examination failed to detect fluid collection. Local status deteriorated with spread of swelling and redness to patient's back and posterior thigh with palpable crepitations (Fig. 1). Computed tomography (CT) scan of chest, abdomen and thighs revealed large collections of air ranging from mid back to popliteal region with necrosis of left gluteal muscle. Emergency surgery was indicated and she was prepared for general anesthesia. Generous incision was placed over the collections of gas and purulent fluid. Extensive debridement of devitalized gluteal muscle was undertaken and the wounds were lightly packed (Fig. 2). Swabs taken isolated CP which confirmed clinical suspicion of clostridial myonecrosis. Antibiotic therapy was corrected to metronidazole and clindamycin. After three successive additional debridements her wounds were treated with negative-pressure wound therapy (NPWT) (VivanoTec®, Paul Hartmann AG, Heidenheim an der Brenz, Germany) (Fig. 3). Edges of the wounds were approximated in a stepwise fashion with secondary sutures (Fig. 4). Patient's general condition and blood tests improved rapidly. Patient was transferred to the Department of Surgery where she was ambulating with the NPWT machine. After six successive NPWT exchanges, almost all of the wounds were sutured and the patient was discharged on the 18th postoperative day. She was followed-up as an outpatient over the next 3 weeks where further improvement of nutritional and local wound status occurred. Finally, all the wounds were sutured and healed without signs of infection (Fig. 5). Full mobility and hip function were restored in a short period of time.

## Discussion

Contamination of traumatic and postoperative wounds by clostridial spores or extremely rarely inoculation via intramuscular drug administration is the gateway for infection. Local wound conditions such as contused or necrotic tissue with low oxidation-reduction potential allows germination of spores. Comorbidity such as diabetes and long-term corticosteroid therapy are additional risk factors. Infection induces muscle necrosis, thrombosis of blood vessels and marked edema which further compromise tissue oxygen supply. Production of gas caused by glucose fermentation helps spreading infection by dissection of fascial and subcutaneous planes. Sole presence of gas inside of tissue or wound does not exclusively means clostridial infection since a number of other bacteria are capable of producing gas such as aerobic gram-negative bacteria (*Escherichia coli*, *Proteus*, *Pseudomonas aeruginosa* and *Klebsiella pneumoniae*). There is no specific marker that proves the clostridial infection, therefore we must seek for early clinical signs in adjunction with laboratory and imaging findings. Blood count reveals signs of serious infection with elevated white blood cells, left shift and elevated CRP level. Immunocompromised patients' reaction to infection is mitigated and might be misleading. Metabolic acidosis and renal failure are common in gas gangrene but it is not specific as it only translates remote tissue injury. The hemoculture rarely catch the offender, however it is essential to eliminate other causes of sepsis. The diagnosis is confirmed after isolation of CP in infected tissue. Imaging is valuable for estimating the extent of the infection and necrosis. As shown in this case, early use of computed tomography (CT) is recommended. A recent study reported 100% sensitivity of CT in detecting necrotizing infection [1]. Ultrasound (US) is still a viable alternative with advantages of being available at bedside and having excellent sensitivity in detecting gas. However, it is highly operator dependent, which may lead to falsely negative findings [2]. The treatment consists of antibiotic therapy and meticulous surgical debridement of all necrotic and nonviable tissues. First line antibiotics are combination of penicillin and clindamycin, but metronidazole can be used alternatively [3]. NPWT is relatively new technology that can aid in the treatment of large wounds with tissue loss. It works through mechanical removal of debris, excessive tissue fluid, pus and bacteria. At the same time dressing keeps a sealed environment, preventing secondary nosocomial infection. One of the main benefits of a negative pressure is improvement of tissue oxygenation through stimulation of capillary perfusion and edema withdrawal [4]. Less edema means less intercellular fluid which shortens the path between the vessels and the target cells. NPWT is routinely applied in chronic wounds and pressure ulcers but it has versatile use such as rapid air collections removal in recalcitrant post-traumatic subcutaneous emphysema [5]. Our case shows efficiency of NPWT on large wound with significant tissue loss, allowing gradual closure in a short time period without the need for skin grafting. Literature search reveals several cases of gas gangrene caused by administration of various drugs via different parenteral routes such as intraarticular, intramuscular and intravenous [[6], [7], [8], [9], [10]]. Applied drugs (steroids, diclofenac, NSAID, crystalloids, vitamin B) do not have common denominator. Routine skin preparation before parenteral application of a drug consists of alcohol or chlorhexidine swabbing which destroys vegetative forms of microorganisms but not spores. Gluteal or pelvic region might pose additional risk for presence of clostridial spores on the neighboring skin due to proximity of anus. Therefore, the culprit is not the injected medicine but direct inoculation of spores, especially in patients with additional risk factors such as diabetes, chronic steroid therapy and immunosuppression.
